# Complementarity of the residue-level protein function and structure predictions in human proteins

**DOI:** 10.1016/j.csbj.2022.05.003

**Published:** 2022-05-06

**Authors:** Bálint Biró, Bi Zhao, Lukasz Kurgan

**Affiliations:** a Institute of Genetics and Biotechnology, Hungarian University of Agriculture and Life Sciences, Gödöllő, Hungary; b Department of Computer Science, Virginia Commonwealth University, Richmond, VA, United States

**Keywords:** Protein structure prediction, Protein function prediction, Intrinsic disorder, Secondary structure, Solvent accessibility, Nucleic acid binding, Evaluation, Meta-prediction, Webserver

## Abstract

Sequence-based predictors of the residue-level protein function and structure cover a broad spectrum of characteristics including intrinsic disorder, secondary structure, solvent accessibility and binding to nucleic acids. They were catalogued and evaluated in numerous surveys and assessments. However, methods focusing on a given characteristic are studied separately from predictors of other characteristics, while they are typically used on the same proteins. We fill this void by studying complementarity of a representative collection of methods that target different predictions using a large, taxonomically consistent, and low similarity dataset of human proteins. First, we bridge the gap between the communities that develop structure-trained vs. disorder-trained predictors of binding residues. Motivated by a recent study of the protein-binding residue predictions, we empirically find that combining the structure-trained and disorder-trained predictors of the DNA-binding and RNA-binding residues leads to substantial improvements in predictive quality. Second, we investigate whether diverse predictors generate results that accurately reproduce relations between secondary structure, solvent accessibility, interaction sites, and intrinsic disorder that are present in the experimental data. Our empirical analysis concludes that predictions accurately reflect all combinations of these relations. Altogether, this study provides unique insights that support combining results produced by diverse residue-level predictors of protein function and structure.

## Graphical abstract

**Figure:**
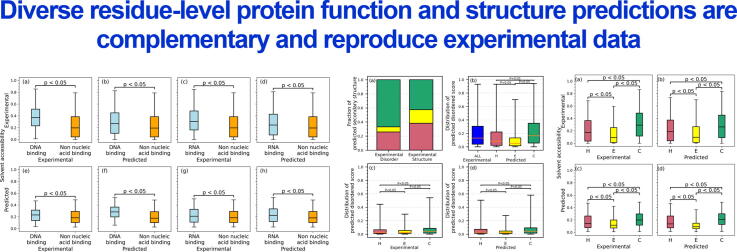

## 1 Introduction

The residue-level annotations, also called 1-dimensional annotations, cover a broad spectrum of structural and functional characteristics of amino acids in protein sequences [1], [2]. They include various structural characteristics, such as secondary structure, solvent accessibility, intrinsic disorder, and flexibility, as well as functional features, such as catalytic, cleavage and post-translational modification sites and residues that interact with proteins and nucleic acids. The amount of the experimental residue-level annotations, which are commonly derived from the structural data available in Protein Data Bank (PDB) [3], lags behind the rapidly growing number of protein sequences. The current version 2021_04 of UniProt covers 225 million sequences and has doubled in size since 2008 [4]. The huge amount of protein sequences that lack the residue-level annotations has motivated the development of hundreds of computational methods that predict these annotations from the sequences. For instance, there are over 60 predictors of the secondary structure [5], [6], [7], over 100 predictors of the intrinsic disorder [8], [9], [10], [11], [12], and close to 40 predictors of the residues that bind nucleic acids [13], [14], [15]. Some of these methods are heavily used, which can be indirectly measured by their citations. For instance, the popular predictors of secondary structure, PSIPRED [16], intrinsic disorder, IUPred [17], and glycosylation and phosphorylation sites, NetPhosK [18], were cited 6338, 2013, and 2002 times, respectively (source: Google Scholar as of February 14, 2022). Results produced by these tools are utilized to produce hypotheses and support experimental investigations. For instance, our DisoRDPbind [19], [20], which predicts residues that interact with proteins and nucleic acids, was recently used to study the SARS-CoV-2 proteome [21], decode functions of genes from pathogenic organisms [22], and investigate the mixed lineage leukemia 4 (MLL4) [23], heat shock factor 1 (Hsf1) [24] and mediator complex subunit 15 (MED15) [25] proteins that are associated with cancer and neurodegenerative diseases. Furthermore, results produced by these predictors for millions of proteins and thousands of organisms are easily accessible via several popular large databases including D^2^P^2^ [26], MobiDB [27] and DescribePROT [28].

Availability of the many sequence-based predictors of the residue-level annotations has spurred numerous studies that survey and compare these tools [1], [2], [5], [6], [7], [8], [9], [10], [11], [13], [14], [15], [29], [30], [31], [32], [33], [34], [35], [36], [37], [38], [39], [40], [41], [42], [43], [44], [45], [46]. A large portion of these studies focuses on the empirical comparative assessment of their predictive performance. These computational tools use predictive models trained and tested using the ground truth generated by experimental methods. They often rely on models produced from training data by machine learning (ML) algorithms. Predictions produced by these models on test data are evaluated against the experimental ground truth and compared across different methods. While predictive performance of the published tools is typically evaluated by the authors in the corresponding publications, these assessments are usually limited in scope (i.e., relatively few methods are compared) and may rely on small test datasets and non-standard test protocols and metrics. Consequently, large scale comparative studies were carried out for some of the predictive targets including prediction of the secondary structure [1], [7], [38], [39], intrinsic disorder [1], [40], [41], [42], [43], solvent accessibility [1], protein interactions [29], and nucleic acids interactions [13], [15], [32], [44]. Moreover, several community assessments were completed. These evaluations are done by independent assessors (i.e., they do not participate as predictors) on blind datasets (i.e., ground truth is unavailable to the predictors) using test protocols and metrics that are agreed on by the corresponding community. For instance, the secondary structure predictors were evaluated as part of the Critical Assessment of Structure Prediction (CASP) between CASP3 and CASP5 [47], the Critical Assessment of Fully Automated Structure Prediction (CAFASP) [48], [49], [50], and the EVAluation of protein structure prediction servers (EVA) [51] community assessment efforts. The disorder predictions were evaluated by the community as part of CASP between CASP5 [52] and CASP10 [53] and recently in the Critical Assessment of protein Intrinsic Disorder (CAID) [41]. The sheer number and scale of these studies demonstrates significant interest in the residue-level predictors. These surveys and assessments provide invaluable insights about the predictors. They summarize and categorize the available methods, quantify and compare their predictive quality, evaluate progress and often suggest future research directions. These insights assist the end user to rationally select the best tools and the developers to appropriately focus their efforts.

However, these studies share certain shortcomings. ***First***, they always analyze and compare predictors that target the same structural/functional characteristic. In other words, relations between different predictions were not assessed while they are biologically relevant. For instance, interaction sites are located on the protein surface, and thus it would be pertinent to investigate whether the predicted interactions agree with the putative solvent accessibility. A special case of this overlooked aspect is the presence of two distinct subcommunities that predict residue-level interactions. This stems from the fact that the corresponding experimental annotations are sourced from two databases: PDB [3] that provides access to the structures of the protein–protein and protein-nucleic acids complexes, and DisProt [54] that stores experimental annotations of the interacting residues that are intrinsically disordered (unstructured) [55], [56]. Correspondingly, one subcommunity develops predictors that focus on the structure-annotated interactions [13], [29] while the other on the interactions in the disordered regions of the protein sequence [8], [57]. Recent work shows that the methods produced by the two subcommunities complement each other for the prediction of the protein-binding residues [58]. The unanswered question is whether this is also the case for the prediction of the interactions with nucleic acids. Altogether, studies that evaluate complementarity of predictors that target different characteristics and that use disorder-trained vs. order-trained data are lacking. ***Second***, the test datasets employed across different studies cover taxonomically different protein chains. This makes it relatively difficult to compare empirical results across studies. Case in point is the fact that very different amounts of intrinsic disorder are found in eukaryotes vs. prokaryotes [59], [60] while the quality of the disorder predictions depends on the amount of disorder [42].

We report results of an investigation that addresses these issues. We specifically focus on analyzing representative tools that predict several popular and different types of structural and functional characteristics including secondary structure, solvent accessibility, intrinsic disorder, and residues interacting with nucleic acids. We do not attempt to compare multiple methods that predict the same characteristic since multitude of studies already offer these results [7], [13], [15], [32], [38], [39], [40], [41], [42], [43], [44], [47], [48], [49], [50], [51], [52], [53]. We curate a taxonomically consistent dataset composed of human proteins that shares low similarity with the training data used to develop the considered predictors. We study whether combining results generated by the structure- and disorder-trained predictors would lead to substantial improvements and whether relations observed using the experimental data for all pairs of the considered characteristics are accurately replicated by the respective predictions. We note that the former analysis is constrained to the binding predictions since the other characteristics (i.e., secondary structure and solvent accessibility) are limited to the structured state and as such cannot be predicted using disorder-trained predictors. Our particular focus on the intrinsic disorder is indirectly motivated by the fact that the results produced by AlphaFold2 [61], the method that arguably disrupted protein structure prediction field, are less accurate to identify intrinsic disorder when compared to the modern disorder predictors [62], [63], such as flDPnn that we use here [64]. This, combined with an easy access to the predictions of secondary structure, solvent accessibility, intrinsic disorder, and nucleic acids interacting residues for millions of proteins in related databases (i.e., D^2^P^2^ [26], MobiDB [27] and DescribePROT [28]), justifies the utility of the various predictions that we study here.

## 2 Materials and methods

### 2.1 Datasets

Past studies assess different types of predictors on taxonomically inconsistent test datasets. Here, we perform the entire assessment on the human proteome. We select this proteome due to its high coverage by the experimental annotations (i.e., by far the highest coverage in PDB and DisProt), allowing us to collect a large amount of benchmark data. We collect the complete protein sequences of the human proteome from UniProt [4]. We remove protein fragments which we identify with the term “Fragment” in the sequence descriptions, resulting in 43,789 protein sequences. We map the PDB structures to the UniProt proteins with the help of the Structure Integration with Function, Taxonomy and Sequences (SIFTS) software [65]. These structures provide the ground truth annotations of the secondary structure, solvent accessibility and protein-DNA and protein-RNA interactions. We exclude short PDB chains that correspond to peptides (30 or fewer amino acids). In cases where the same UniProt sequence is covered by multiple PDB chains, we select the longest PDB chain to cover a given portion of the UniProt sequence. In case of a tie we pick the chain with the best structure resolution. Consequently, we find 5,133 UniProt sequences that include structural information sourced from 6,417 PDB chains. Moreover, we supplement these annotations with the experimental data on 790 intrinsically disordered human protein that we collect from DisProt [54].

Next, we check the collected proteins against the training datasets of the considered predictors to ensure that the benchmark sequences share appropriately low similarity. We obtain the training sets of the five predictors (ASAquick, PSIPRED, flDPnn, DisoRDPbind and DRNApred; selection of these methods is explained in Section 2.3) and align them with the proteins from the combined set of 5,133 PDB-annotated and 790 DisProt-annotated human proteins using BLAST with 25% sequence similarity [66]. The resulting 2,535 PDB-annotated and 318 DisProt-annotated sequences that share <25% similarity to the training proteins constitute our test dataset. We combine these two protein sets, which results in the test dataset composed of 2,629 human proteins that we use to investigate the complementarity of the considered five predictors. Moreover, we use the remaining 2,598 PDB-annotated and 472 DisProt-annotated human sequences that share >25% sequence similarity with the training datasets to empirically train and validate a neural network model, which we discuss in section 2.4. Combining the 2,598 PDB-annotated and 472 DisProt-annotated proteins results in 2,713 human sequences.

### 2.2 Collection of experimental annotations

We extract the experimental solvent accessibility and secondary structure directly from PDB structures. We run popular DSSP program [67] to collect the 8-state secondary structure and the absolute solvent accessibility for each residue in the 6,417 PDB chains. We convert the 8-state secondary structure to the 3-state secondary structure using the encoding applied by the predictor that we employ [16], which is consistent with recent assessments [7]. More specifically, H and G states are converted to helix (H), E and B to strand (E), and the other states to coil (C). We also normalize the absolute solvent accessibility using the residue-specific factors from ref. [68] to obtain the relative solvent accessibility. Similar to the secondary structure, this ensures that the ground truth is compatible with the corresponding predicted characteristics. Moreover, we collect the experimental intrinsic disorder from DisProt using its ontology and from PDB using published approaches [53], [69].

We apply BioLip, a frequently updated semi-manually curated database of protein–ligand interactions extracted from the PDB structures [70], to collect the annotations of the nucleic acid binding residues. We map the BioLip’s annotations into the human UniProt sequences and find 3,557 DNA-binding residues in 175 DNA-binding proteins (7.4% of residues in these proteins) and 2,368 RNA-binding residues in 106 RNA-binding proteins (6.4% of residues in these proteins). We also collect annotations of the nucleic acid binding residues from DisProt and identify 3,663 DNA-binding residues in 41 DNA-binding proteins (18.8% of residues in these proteins) and 781 RNA-binding residues in 7 RNA-binding proteins (25.8% of residues in these proteins). Furthermore, we collect a dataset of the non-nucleic acid binding human proteins, which is necessary to assess methods that predict interactions with nucleic acids. First, we identify a comprehensive collection of 3,638 nucleic acid binding proteins by integrating data from multiple resources. We collect the DNA-binding proteins from BioLip, 3D-footprint [71], CIS-BP [72], JASPAR [73], HumanTF2 [74], SMiLE-seq [75], animalTFDB [76], and using gene ontology (GO) terms [77] in UniProt. We find the RNA-binding proteins from BioLip, ATtRACT [78], RBPDB [79], and using the GO terms in UniProt. Next, we remove human proteins that share over 30% similarity with any of the 3,638 nucleic acid binding proteins, which we measure with BLAST [66], [80]. We intersect the resulting 24,435 human proteins with the sequences for which we collect experimental data and use the corresponding common subset as the non-nucleic acid binding proteins.

### 2.3 Collection of residue-level structure and function predictions

The prior assessments concentrate on comparing multiple predictors that address the same structural or functional characteristic [7], [13], [15], [32], [38], [39], [40], [41], [42], [43], [44], [47], [48], [49], [50], [51], [52], [53]. We intentionally avoid repeating this type of analysis given the abundance of the available results. We instead analyze several different types of commonly performed predictions including predictions of the solvent accessibility, secondary structure, intrinsic disorder, and RNA- and DNA-binding residues. We select a representative method for each of these characteristics that satisfies the following three requirements: 1) they are computationally efficient to be able to process our large protein set, i.e., runtime <10 s per protein; 2) they have implementations or webservers that facilitate large-scale predictions; and 3) they were published in reputable journals. The five selected predictors are summarized in Table 1. We chose ASAquick [81], fast predictor of the solvent accessibility which secures predictive performance that is competitive with slower, state-of-the-art predictors [82]. We normalize the ASAquick’s outputs the same way as the DSSP-derived solvent accessibility to collect the putative relative solvent accessibility. We picked PSIPRED [16], [83], the most popular secondary structure predictor that ranked among the most accurate predictors in multiple assessments [7], [84]. We utilize the single-sequence version of PSIPRED to scale to the large size of our dataset. We select flDPnn for the disorder prediction [64]. This method is the fastest among the most accurate disorder predictors that were recently evaluated in CAID [41], [85].

**Table 1:** Summary of the selected predictors of the residue-level structure and function predictions.

Prediction target	Predictor name	Predictive model	Availability	Website
Solvent accessibility	ASAquick	Neural network	Code	https://mamiris.com/software.html
Secondary structure	PSIPRED	Neural network	Code and Webserver	https://bioinf.cs.ucl.ac.uk/psipred/
Intrinsic disorder	flDPnn	Deep neural network	Code and Webserver	https://biomine.cs.vcu.edu/servers/flDPnn/
Nucleic acid binding for disordered regions	DisoRDPbind	Logistic regression	Webserver	https://biomine.cs.vcu.edu/servers/DisoRDPbind/
Nucleic acid binding for structured regions	DRNApred	Logistic regression	Webserver	https://biomine.cs.vcu.edu/servers/DRNApred/

Moreover, we cover predictions of the nucleic acids-binding residues with two methods that represent the corresponding two subcommunities: one that focuses on the structure-based interactions and the other that develops predictors of interactions for the disordered regions. We select methods that predict both DNA-binding and RNA-binding residues and which provide accurate results by minimizing a recently identified cross-prediction issue [13], [15], [86]. The cross-prediction means that predictors of the DNA-binding residues also predict a significant number of residues that bind other ligands (RNA and proteins) as DNA-binding, effectively predicting binding residues irrespectively of the underlying ligand. We choose DisoRDPbind [19], [20], [87], which is fast and provides accurate and cross-prediction reduced results [19], [58]. This tool was recently ranked second-best in the prediction of the interacting disordered residues in the CAID experiment [41], second only to ANCHOR2 [88] that predicts protein-binding residues. Moreover, we select DRNApred [89] that predicts DNA and RNA binding residues using models trained from the PDB structures, and which was developed to minimize the cross-predictions [86], [89]. The residue-level experimental annotations and predictions of the solvent accessibility, secondary structure, DNA-binding residues, RNA-binding residues and intrinsic disorder are available in Supplementary Dataset S1 (5,133 PDB-annotated human sequences) and Supplementary Dataset S2 (790 DisProt-annotated human sequences) at https://biomine.cs.vcu.edu/datasets/1Dassessment/. Furthermore, that page provides access to the Supplementary Dataset S1-1 (2,535 PDB-annotated human sequences) and Supplementary Dataset S2-1 (318 DisProt-annotated human sequences) that include predictions and experimental annotations for the datasets of human proteins that share low similarity to the training data used to develop the considered here predictors, which we discuss in Section 2.1.

We briefly summarize predictive performance of the considered predictors on the 2,629 test proteins that share <25% similarity with their training proteins. The Pearson correlation coefficient for the solvent accessibility predictions from ASAquick is 0.52, which is comparable to the previously reported value of 0.64 [90]. The Q_3_ (3-state accuracy) of the secondary structure predictions produced with the single-sequence PSIPRED is 67.6, which is nearly the same as the Q_3_ = 66.8 that was measured in an earlier study [91]. CAID that uses the DisProt-annotated proteins reports AUC (area under the receiver operating characteristics curve) = 0.81 for flDPnn [41], which is on par with AUC = 0.79 that flDPnn secures on the 318 DisProt-annotated human test proteins. We assess DisoRDPbind and DRNApred using the DNA-/RNA-binding proteins and non-nucleic acid binding proteins from the test dataset. The results that we report in Section 3.1 reveal that DisoRDPbind secures AUC = 0.65 for the DNA binding and AUC = 0.62 for the RNA binding. This is consistent with a recent assessment where DisoRDPbind’s AUC are 0.67 and 0.60, respectively [92]. Similarly, we report AUC = 0.68 for DNA binding and AUC = 0.60 for RNA binding for DRNApred, while the previously published results are 0.68 and 0.65, respectively [89]. Overall, we find that the predictive performance assessed on the human proteins is relatively similar to the results that were reported in the past studies for the same predictors. These results validate quality of the experimental and putative data that we collected.

### 2.4 Development of the MetaNucBind model

The current predictors of the protein and nucleic acids binding residues [8], [13], [15], [29], [32], [44], [57], [93], [94], [34], [95] are clustered into two distinct groups based on the source of training data used to derive their predictive models: structures of the protein–protein/nucleic acid complexes that are sourced from PDB [3] (i.e., structure-trained) vs. the disordered binding regions that are sourced from DisProt [54] (i.e., disorder trained). Interestingly, the corresponding two prediction subcommunities test their methods on the datasets that use the same source data type. We recently evaluated predictors of the protein-binding residues on the disorder and structure annotated data and found that combining the two types of methods produces substantially more accurate predictions [58]. This suggests that the structure- and disorder-trained predictors of protein-binding residues complement each other and motivates us to investigate complementarity of predictors of the nucleic acid binding residues.

We develop MetaNucBind, a neural network-based meta predictor that uses the RNA- and DNA-binding predictions from the disorder-trained DisoRDPbind and the structure-trained DRNApred to predict the combined set of disordered and structured binding residues. We utilize a small deep feed-forward neural network (FFNN) that uses a sliding window of predictions from DisoRDPbind and DRNApred as the input to predict the DNA-binding and RNA-binding propensities for the residue in the middle of the window. We implement this network using python 3.8 with Kares (2.4.0), Scikit-learn (0.24.2), Numpy (1.18.5), and Tensorflow (2.3.0) libraries.

We parametrize the FFNN model, i.e., select the number of hidden layers and the window size = {1, 3, 5, and 7}, using training and validation data extracted from the set of 2,713 proteins that share high similarity with the training proteins of the selected predictors, which we discuss in Section 2.1. We randomly select 70% of these proteins to form a training dataset and the remaining 30% to establish a validation dataset. The training, validation and test datasets, including the experimental residue-level annotations of the DNA- and RNA-binding, are available as the Supplementary Datasets S3, S4, and S5, respectively, at https://biomine.cs.vcu.edu/datasets/1Dassessment/. The use of different window sizes evaluates whether predictions for the adjacent residues would be useful to more accurately determine prediction for the central residue. We select the parameters that results in the maximal AUC on the validation dataset. The resulting model uses the window size of 5 and is composed of three hidden layers with 6, 4 and 2 nodes, and the output layer with one node that produces the DNA- and RNA-binding propensities. We observe that the use of windows produces marginal improvements in the predictive quality, i.e., AUC increases by 0.01 for the DNA binding prediction and by 0.005 for the RNA binding prediction when comparing FFNNs that do not use a window (window size = 1) with those that use the window of size 5. This suggests that the use of the predictions that are adjacent in the sequence does not provide substantial improvements for the prediction of the nucleic acid binding residues.

## 3 Results and discussion

### 3.1 Predictors of the nucleic acid interacting residues trained on the disordered and structured data complement each other

Using the DNA-/RNA-binding proteins and non-nucleic acid binding proteins from the low-similarity test dataset discussed in Section 2.1, we evaluate the disorder-trained DisoRDPbind [19], the structure-trained DRNApred [89], and the MetaNucBind that combines their predictions using the deep FFNN model. We also explore several simple approaches to combine the two predictions including taking the minimum, maximum, and the average of the normalized outputs from the two predictors, DisoRDPbind and DRNApred. We summarize these results on the test dataset in Table 2. We quantify predictive performance with the commonly-used AUC; the corresponding ROC curves are in the Supplementary Fig. S1. Moreover, we provide a selection of metrics for the binary predictions that rely on a threshold to binarize the predicted numeric propensities. We ensure that the binary predictions are standardized across methods by setting a threshold that produces consistent prediction rate, which in turn facilitates direct side-by-side comparisons. We compute sensitivity at fixed false positive rates (FPRs) of 0.2 and 0.3, and specificity at fixed true positive rates (TPR, which is the same as sensitivity) of 0.4 and 0.5.

**Table 2:** Assessment of predictions of the RNA-binding and DNA-binding residues on the low-similarity test dataset composed of the DNA-binding, RNA-binding and non-nucleic acid binding human proteins. The evaluation covers MetaNucBind, the structure-trained DRNApred, the disorder-trained DisoRDPbind, and four combinations of their predictions where “Min”/”Max”/”Average” are the minimal/maximal/average value of the two predictions.. We assess whether differences in predictive quality between the most accurate MetaNucBind and the other methods are robust to different datasets, i.e., we repeat tests 50 times using randomly selected subsets of 50% of test proteins. We assess significance of differences in the AUC, sensitivity and specificity scores using the *t*-test if the underlying data are normal; otherwise, we use the Wilcoxon signed-rank test; we test normality with the Anderson-Darling test at the 0.05 significance. * denotes that the difference when compared to MetaNucBind is statistically significant at *p*-value < 0.05 significance, ^∼^ means that the difference is not statistically significant (*p*-value ≥ 0.05). The best results for a given metric are shown in bold font.

Target interaction	Predictor type	Predictor name	AUC	Sensitivity at FPR = 0.2	Sensitivity at FPR = 0.3	Specificity at TPR = 0.4	Specificity at TPR = 0.5
DNA-binding	Disorder-trained	DisoRDPbind	0.654*	0.418*	0.530*	0.817*	0.731*
	Structure-trained	DRNApred	0.679*	0.499*	0.601*	0.859*	0.798*
	Combination	Min	0.651*	0.445*	0.541*	0.839*	0.736*
		Max	0.714*	0.513*	0.632*	0.864*	0.807*
		Average	0.717*	0.524^∼^	0.639^∼^	**0.878^∼^**	0.816^∼^
		MetaNucBind	**0.722**	**0.529**	**0.643**	0.876	**0.819**
RNA-binding	Disorder-trained	DisoRDPbind	0.619*	0.344*	0.457*	0.758*	0.666*
	Structure-trained	DRNApred	0.604*	0.374*	0.440*	0.760*	0.638*
	Combination	Min	0.598*	0.321*	0.428*	0.726*	0.634*
		Max	0.692*	0.458∼	**0.585^∼^**	0.831^∼^	0.765^∼^
		Average	0.700^∼^	0.452^∼^	0.582^∼^	**0.838^∼^**	0.766^∼^
		MetaNucBind	**0.704**	**0.454**	0.578	0.835	**0.770**

We find that the structure-trained and disorder-trained predictors secure results that are consistent with their published predictive performance, with AUC ranging between 0.604 and 0.679; see details in Section 2.3. Table 2 shows that the minimum-based combination performs rather poorly, with AUCs lower than the AUCs of the input predictors. This can be explained by the fact that the two input predictions are trained to generate high propensities to identify two distinct collections of binding residues (structure vs. disorder trained), and thus selecting a minimum effectively reduces the number of predicted binding residues. This is why the corresponding sensitivity values at 0.2 FPR are lower (0.445 for the DNA binding and 0.321 for the RNA binding) when compared to the sensitivity secured by the input predictors (0.499 for DRNApred’s DNA binding and 0.374 for DisoRDPbind’s RNA binding). The same is true when using the other binary metrics. The max-based and the average-based combinations produce similar results, with the average having a slight edge. The average-based consensus outperforms the results of the input predictors by the wide margin, with AUC = 0.717 vs. 0.654 and 0.679 for the DNA binding, and with AUC = 0.700 vs. 0.604 and 0.619 for the RNA binding. The corresponding sensitivity at 0.2 FPR improves by a similarly large margin, from 0.499 to 0.524 for the DNA binding, and from 0.374 to 0.452 for the RNA binding. Overall, the reduction in sensitivity when using the minimum-based approach coupled with the increase in sensitivity when using the average- and maximum-based combination suggests that the binding residues predicted by the two methods share a limited amount of overlap and complement each other.

The more sophisticated MetaNucBind model provides a modest amount of improvements over the average-based consensus, which can be attributed to the use of the neural network. However, the increase in the performance over the results produced by the disorder-trained and structure-trained predictors is substantial. For the DNA binding prediction, MetaNucBind secures AUC = 0.722 and sensitivity = 0.529 at 0.2 FPR, compared to AUC = 0.679 and sensitivity = 0.499 for the best input predictor. Similarly, the MetaNucBind’s AUC and sensitivity at 0.2 FPR are 0.704 and 0.454, respectively, for the RNA binding predictions vs. 0.619 and 0.374 for the best input predictor. These improvements are statistically significant for both DNA-binding and RNA-binding (*p*-value < 0.05). Altogether, these results reveal that the structure- and disorder-trained methods generate complementary predictions, which when combined together produce significantly higher predictive quality. This is consistent with the conclusions that were reported in the context of the prediction of the protein-binding residues [58]. Moreover, we find that the improvements are largely attributed to the complementary nature of the structure-trained and disorder-trained predictions (i.e., large increase for the average or maximum-based combinations vs input predictors), rather than to using a sophisticated model to combine these predictions (i.e., we note the modest improvements of MetaNucBind vs. the average-based model).

We provide the MetaNucBind predictor as a free and convenient webserver located at https://biomine.cs.vcu.edu/servers/MetaNucBind/. This page collects the FASTA-formatted sequence of the input protein and an optional email address. We send link to the results to that email after the predictions are completed. The users are also directed to the results in the browser window. The prediction process is fully automated and completed on the server side. We provide the results in a parsable text file that includes the sequence of the query protein, the putative propensities of DNA-binding and RNA-binding, and the binary predictions of the putative DNA-binding residues and RNA-binding residues at the FPR of 0.2 and 0.3. The MetaNucBind’s website also provides access to the training, validation and test datasets used in this project.

### 3.2 Predictions accurately replicate relations between structural and functional characteristics

The residue-level structural and functional characteristics are inherently related with each other. For instance, binding residues are expected to have high solvent accessibility. We empirically identify relations between different experimentally measured residue-level annotations for the six possible combinations of the considered four characteristics: intrinsic disorder, secondary structure, solvent accessibility and RNA/DNA interaction sites. Next, we investigate whether these relations are correctly replicated by the corresponding predictions in order to find whether the different types of predictions provide complementary information for the same protein. These experiments rely on the test dataset with the 2,629 sequences that shares low (<25%) similarity with the training datasets of the considered here predictors.

**Fig. 1:**
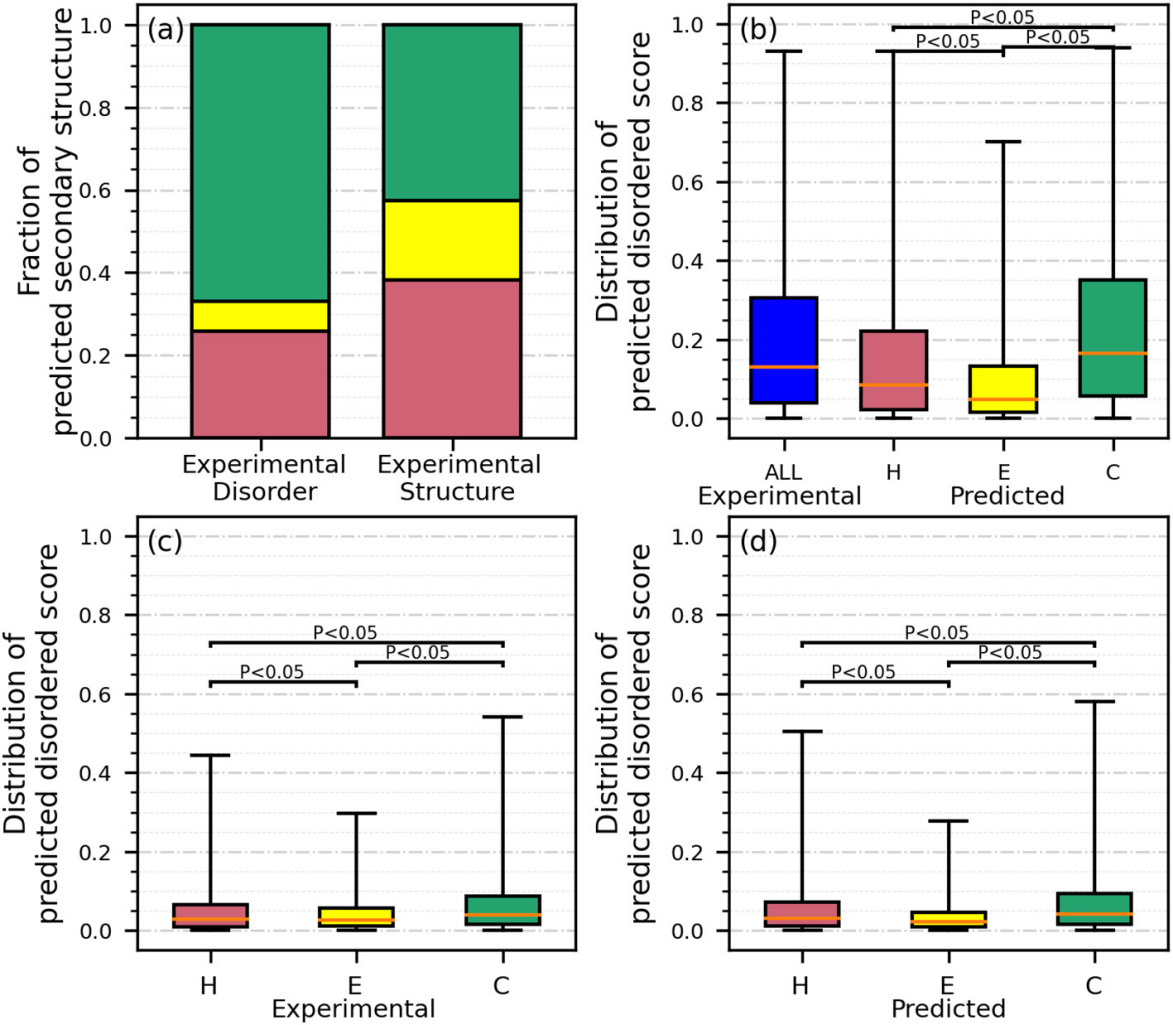
Intrinsic disorder and secondary structure in the part of the low-similarity test dataset that has the corresponding experimental data. Panel (a) contrasts putative secondary structures between the experimentally verified disordered vs. structured residues. Panel (b) summarizes the disorder predictions for the experimentally verified disordered residues (in blue) and for these experimentally verified disordered residues grouped by their predicted secondary structure. The two bottom panels compare the disorder predictions for native structured residues where their secondary structure is based on the experimental data (panels (c)) and based on the prediction (panels (d)). The color-coded box plots (blue for disordered residues, red for helix, yellow for strand, and green for coil) represent distributions of the predicted disorder propensities using the 5th (bottom whisker), 25th, 50th, 75th and 95th (top whisker) percentiles. We assess significance of differences in the disorder propensity values between residue sets identified on the *x*-axis using the *t*-test if the underlying data are normal; otherwise, we use the Wilcoxon signed-rank test; we test normality with the Anderson-Darling test at the 0.05 significance. The corresponding *p*-values are at the top of the box plots. (For interpretation of the references to color in this figure legend, the reader is referred to the web version of this article.)

Disordered protein regions carry out a diverse range of cellular functions while they lack a well-defined equilibrium structure under physiological conditions [55], [96]. Bioinformatics studies estimate that between 40 and 50% of the human proteins have disordered regions [26], [60], [97], [98]. While disordered proteins/regions are unstructured in isolation, some of them fold into well-defined structures upon binding with a target molecule [99], [100]. This suggests these regions possess propensity to form structure and raises a question whether and how experimental annotations and predictions of intrinsic disorder and secondary structure are related. Fig. 1 summarizes the corresponding results on the test dataset. Fig. 1(a) compares proportions of predicted secondary structures between the experimentally verified disordered vs. structured residues. The proportions for the native structured residues are 0.38 for helix, 0.21 for strand and 0.41 for coil and they substantially shift in favor of the most structurally flexible coil conformation for the native disordered residues, i.e., 0.26 for helix, 0.08 for strand and 0.66 for coil. This suggests that the secondary structure predictions are sensitive to the location of the experimentally annotated disordered regions. The helix and strand predictions among the native disordered regions can be justified by the fact that a substantial portion of disordered regions that fold upon binding includes these secondary structure states [101], [102], [103]. Moreover, these folded states are significantly enriched in the helical conformations when compared to the strands [102], which agrees with our observations. Fig. 1(b) investigates disorder predictions for the experimentally annotated disordered residues while Fig. 1(c) and (d) analyze these predictions for the native structured residues. As expected, we find that the predicted propensities for disorder are much higher for the native disordered residues (Fig. 1(b)) than for the structured residues (Fig. 1(c) and (d)). Moreover, Fig. 1(c) demonstrates that the highest putative disorder propensities are for the native coil residues, followed by helices and by strands, with all corresponding differences being statistically significant (*p*-value < 0.05). Importantly, these relations are accurately reproduced when using predicted secondary structure (Fig. 1(d)), and even when making predictions for the native disordered residues (Fig. 1(b)). Altogether, our analysis implies that disorder and secondary structure predictions are in good agreement with each other and with the underlying experimental data.

Next, we investigate the relation between intrinsic disorder and solvent accessibility (Supplementary Figure S2). We note that disordered regions lack well-defined structure and their solvent accessibility cannot be measured. However, we compare the putative solvent accessibility between the experimental disorder (Supplementary Figure S2(a)) and the putative disorder (Supplementary Figure S2(b)). We find that disordered regions have on average significantly higher putative solvent accessibility (*p*-value < 0.05) and this relation holds true irrespective of whether we use experimental or predicted disorder. This suggests that putative solvent accessibility is a viable marker that can be used to identify native disordered regions.

**Fig. 2:**
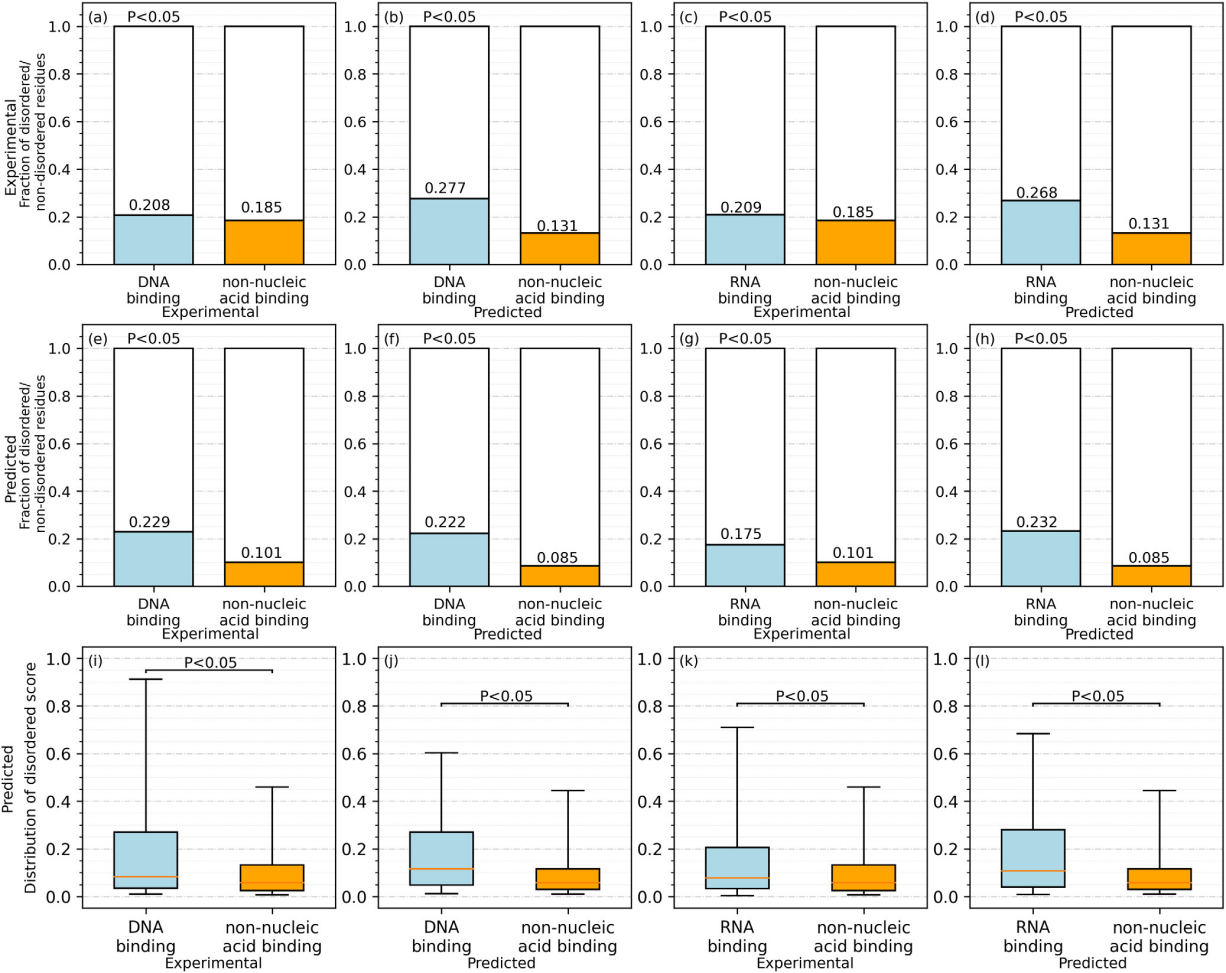
Intrinsic disorder for the DNA-/RNA-binding and non-nucleic acid binding residues in the part of the low-similarity test dataset that has the corresponding experimental data. The six panels on the left (a, b, e, f, i, and j) focus on the DNA-binding residues. The six panels on the right (c, d, g, h, k, and l) show results for the RNA-binding residues. The first and third columns of panels (a, e, i, c, g, and k) show results for the experimental DNA-/RNA-binding and non-nucleic acid binding residues, while the second and fourth columns of panels (b, f, j, d, h, and l) summarize results for the putative DNA-/RNA binding and non-nucleic acid binding residues generated by MetaNucBind. The color-coded bar plots (a, b, c, d, e, f, g, and h) represent the fraction of the disordered residues on binding (blue) and non-nucleic acid binding residues (orange), and the white bars stand for the fraction of non-disordered residues among the binding and non-nucleic acid binding residues. The color-coded box plots (blue for binding, orange for non-binding) represent distributions of the disorder propensity scores using the 5th (bottom whisker), 25th, 50th, 75th, and 95th (top whisker) percentiles. We assess whether differences in the fractions of disorder between DNA-/RNA-binding and non-nucleic acid binding residues are robust to different datasets, i.e., we repeat tests 100 times on randomly selected subsets of 20% of test proteins. We assess the significance of differences in the fraction of disordered residues/disordered scores between DNA-/RNA-binding and non-nucleic acid binding residues using the *t*-test if the underlying data are normal; otherwise, we use the Wilcoxon signed-rank test; we test normality with the Anderson-Darling test at the 0.05 significance. The corresponding *p*-values are shown inside the plots. (For interpretation of the references to color in this figure legend, the reader is referred to the web version of this article.)

Fig. 2(a) and (c) show that the fractions of experimental disordered residues among the native DNA-binding and RNA-binding residues are 0.208 and 0.209, respectively, which are around 12% higher than 0.185 that we measure for the non-nucleic acid binding residues. These differences are statistically significant (*p*-value < 0.05). This observation is supported by several studies that suggest that intrinsic disorder is substantially enriched among the DNA-binding and RNA-binding proteins [104], [105], [106], [107], [108], [109], [110]. Fig. 2(b) and (d) also reveal large and statistically significant differences in the amounts of the experimental disordered residues among the residues grouped based on the predicted DNA/RNA-binding annotations (*p*-value < 0.05). Moreover, Fig. 2(e) and (g) demonstrate that the putative disordered residues are significantly more abundant among the native DNA-/RNA-binding residues than among the native non-nucleic acid binding residues (*p*-value < 0.05). This concurs with a recent study that finds that disorder predictions are accurate for the nucleic acid-binding proteins [40]. Most importantly, we find that the fractions of the putative disordered residues among the predicted DNA/RNA-binding residues follow the same relation as for the experimental data, including the fact that differences are large and statistically significant (*p*-value < 0.05) (Fig. 2(f) and (h)). We further explore these relations based on the putative propensity of disorder, which is generated by most of the disorder predictors. In agreement with the results that consider fraction of the disordered residues, we find that the putative propensities are much higher among the native DNA/RNA-binding residues (Fig. 2(i) and (k); *p*-value < 0.05), as well as among the predicted DNA-/RNA-binding residues (Fig. 2(j) and (l); *p*-value < 0.05). To sum up, we observe that DNA-/RNA-binding residues are substantially enriched in the intrinsic disorder compared to the non-nucleic acid binding proteins, and that these relations are reflected by both experimental and predicted data.

**Fig. 3:**
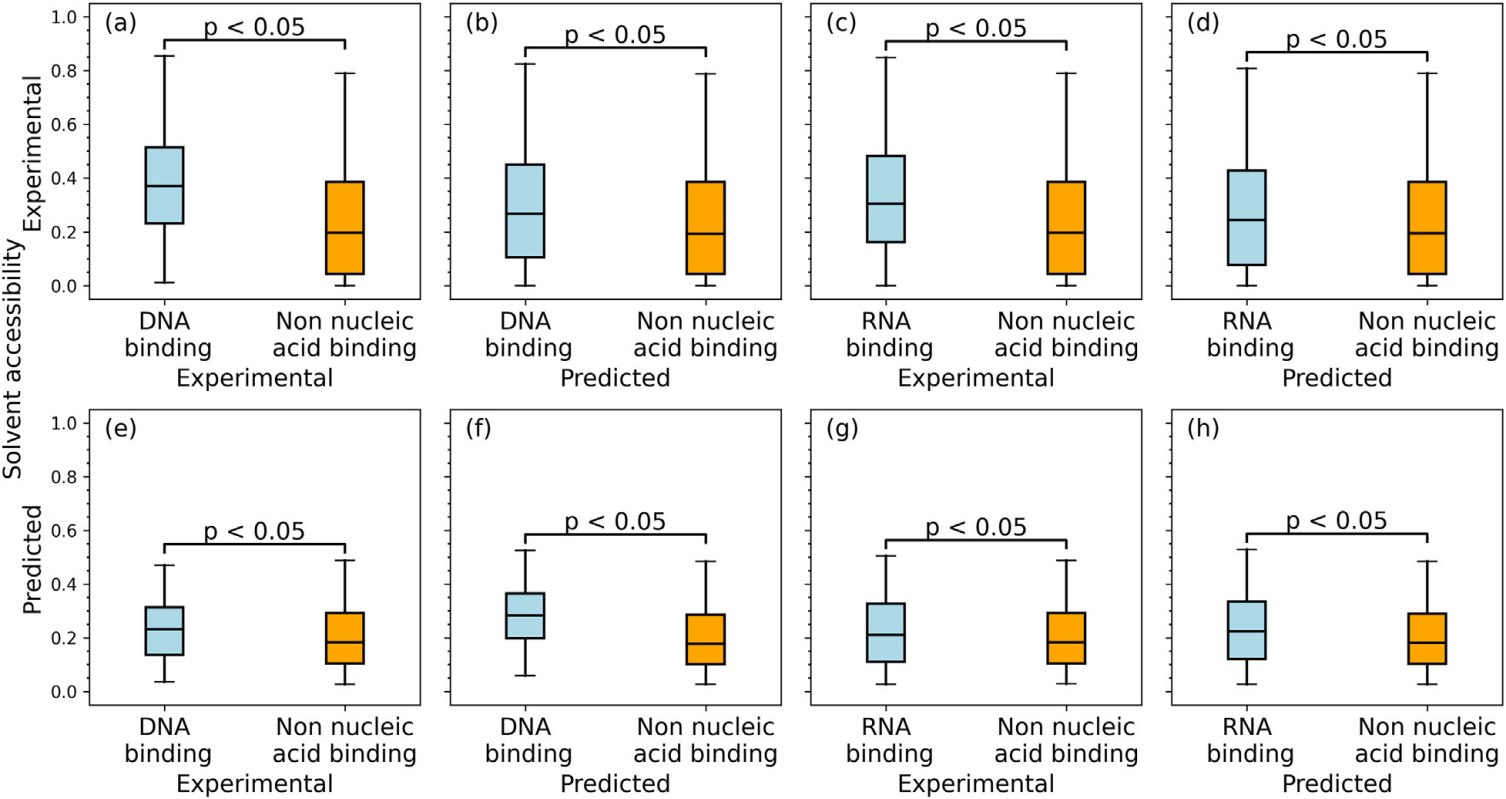
Solvent accessibility for the DNA-/RNA-binding and non-nucleic acid binding residues in the part of the low-similarity test dataset that has the corresponding experimental data. The four panels on the left (a, b, e, and f) focus on the DNA-binding residues. The four panels on the right (c, d, g, and h) show results for the RNA-binding residues. The upper (lower) row of panels shows the experimental (predicted) solvent accessibility. The first and third columns of panels (a, e, c, and g) show the experimental DNA-/RNA-binding and non-nucleic acid binding residues, while the second and fourth columns of panels (b, f, d, and h) give the putative DNA-/RNA binding and non-nucleic acid binding residues generated by MetaNucBind. The color-coded box plots (blue for binding, orange for non-binding) represent distributions of the solvent accessibility values using the 5th (bottom whisker), 25th, 50th, 75th and 95th (top whisker) percentiles. We assess significance of differences in the solvent accessibility values between DNA-/RNA-binding and non-nucleic acid binding residues using the *t*-test if the underlying data are normal; otherwise, we use the Wilcoxon signed-rank test; we test normality with the Anderson-Darling test at the 0.05 significance. The corresponding *p*-values are at the top of the box plots. (For interpretation of the references to color in this figure legend, the reader is referred to the web version of this article.)

Residues that interact with nucleic acids typically localize on the protein surface and thus their solvent accessibility should be higher when compared to the other amino acids [111], [112], [113], [114]. Fig. 3(a) and (c) show that experimental solvent accessibility of the native DNA-/RNA-binding residues is indeed higher than for the non-nucleic acid binding residues (*p*-value < 0.05), confirming observations from the literature. Fig. 3(b) and (d) reveal that the differences in the experimental solvent accessibility between residues grouped based on the predicted binding annotations are also large and statistically significant (*p*-value < 0.05) and consistent with the results based on the experimental annotations of interactions. This implies that the underlying predictions are accurate, which in turn is supported by the past empirical assessments of these methods [19], [89]. Furthermore, Fig. 3(e) and (g) demonstrate that the putative solvent accessibility is much higher for the experimentally annotated binding residues (*p*-value < 0.05), suggesting that the solvent accessibility predictions are useful in differentiating nucleic acid interacting vs. non-interacting residues. The key finding, which stems from Fig. 3(f) and (h), is that the predicted solvent accessibility for the predicted binding residues maintains the same relations as we observe using the experimental data.

**Fig. 4:**
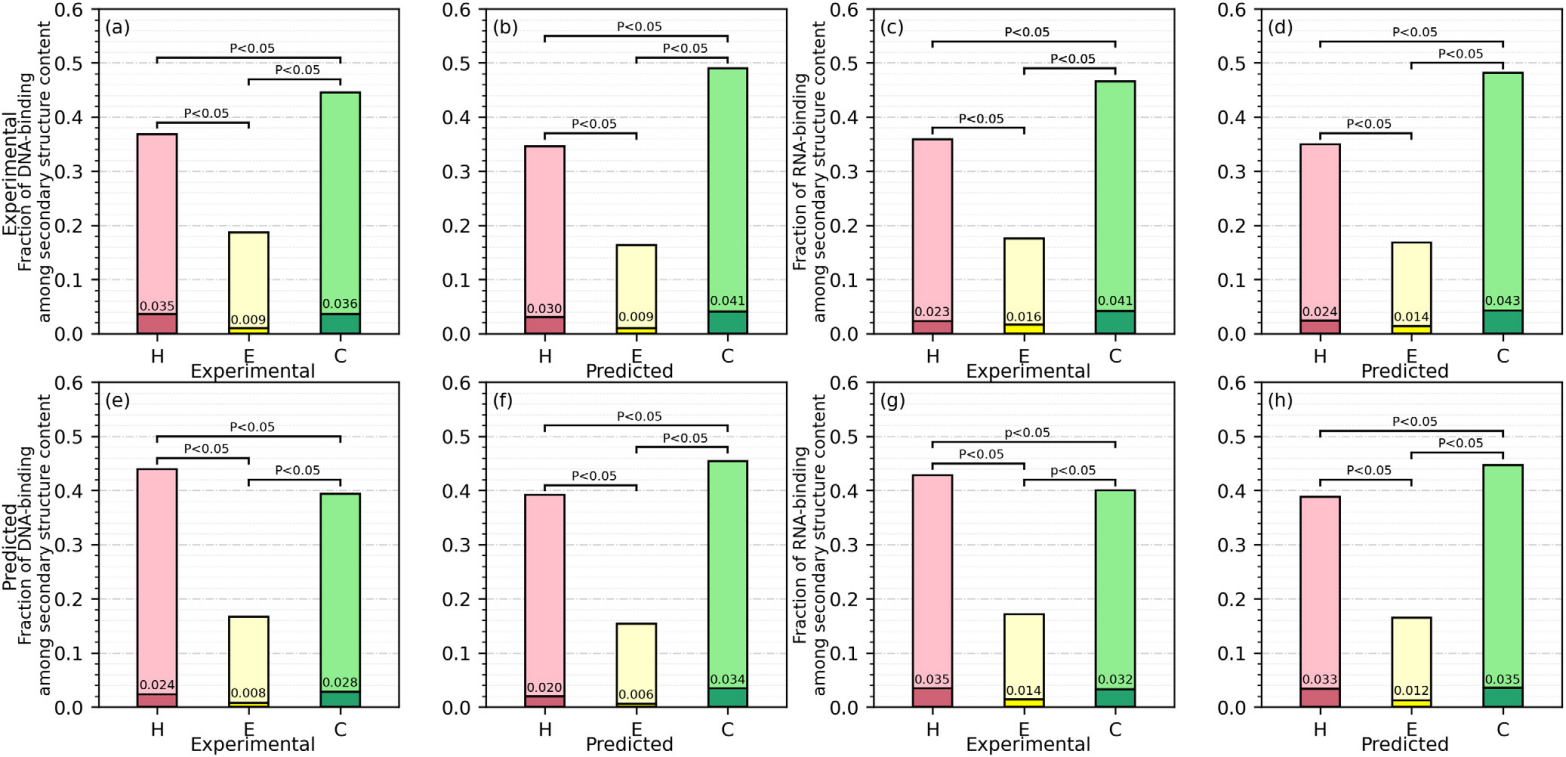
Secondary structure for the DNA-/RNA-binding proteins in the part of the low-similarity test dataset that has the corresponding experimental data. The four panels on the left (a, b, e, and f) focus on the DNA-binding residues. The four panels on the right (c, d, g, and h) show results for the RNA-binding residues. The color-coded bars give the fractions of the secondary structures for residues in the DNA/RNA-binding proteins (light red for helix, light yellow for strand, and light green for coil). The dark-colored areas inside the bars provide relative fractions of the DNA-/RNA-binding residues among the residues grouped by their secondary structures, i.e., DNA/RNA-binding residues among the residues in the helix (in dark red), strand (in dark yellow), and coil (in dark green) conformations. The dark colored areas in the upper row of panels show the relative fractions of the experimentally annotated DNA/RNA-binding residues while the lower row of panels displays these data for the DNA-/RNA-binding residues predicted by MetaNucBind. We assess whether differences in the relative fractions of DNA/RNA-binding residues between residues in different secondary structure states (helix vs. coil, helix vs. strand and strand vs. coil) are robust to different datasets, i.e., we repeat tests 100 times on randomly selected subsets of 20% of test proteins. We assess the significance of differences in relative fractions of DNA-/RNA-binding residues between residues that have different secondary structures (helix vs. coil, helix vs. strand and strand vs. coil) using the *t*-test if the underlying data are normal; otherwise, we use the Wilcoxon signed-rank test; we test normality with the Anderson-Darling test at the 0.05 significance. The corresponding *p*-values are included at the top of the box plots. (For interpretation of the references to color in this figure legend, the reader is referred to the web version of this article.)

We investigate relation between the DNA-/RNA-binding residues and secondary structures in Fig. 4. The fractions of residues in each secondary structure state for the DNA-binding and RNA-binding proteins are shown using the light-colored bars. These results show that the DNA/RNA-binding proteins are enriched in the coil and helix conformations, which together cover over 80% of their sequences, irrespective whether the experimental or putative annotations are used. We also calculate relative fractions of the DNA-binding and RNA-binding residues in each secondary structure state for the DNA-binding and RNA-binding proteins. These values are shown inside the bars and represented using the dark-colored areas. Fig. 4(a) and (c) display the results based on the experimental data. We find that the relative fractions of DNA-binding residues (Fig. 4(a)) in the coil and helix conformation are 0.036 and 0.035, respectively, which is about 4 times higher that the relative fraction of 0.009 in the strand conformation. Similarly, the relative fractions of RNA-binding residues (Fig. 4(c)) in the coil, helix, and strand conformation are 0.028, 0.024, and 0.008, respectively. When compared to the overall rates of the secondary structures shown with the light-colored bars, this suggests that the nucleic acid binding residues are disproportionally depleted among the strand residues. We assess significance of differences in the relative amounts of the DNA/RNA-binding residues between any two secondary structure states and observe that the relative fractions in the coil conformation are statistically higher than in the helix conformation, and in coil/helix conformation are statistically higher than in the strand conformation (*p*-value < 0.05). The enrichment in the helical conformations is supported by studies of coiled-coils motifs in the DNA-/RNA-binding proteins [115], [116]. Fig. 4(b) and (d) compare the relative fractions of native DNA-/RNA-binding residues between different predicted secondary structures. The corresponding relations are consistent with the observation from the experimental data, including the statistical significance (*p*-value < 0.05). This indicates that the predicted secondary structures are relatively accurate, agreeing with the past favorable benchmark results of the PSIPRED method [7], [84]. Fig. 4(e) shows that the relations between putative DNA-binding annotations and the experimental secondary structures replicates the relations between experimental data. However, the relative fraction of the predicted RNA-binding residues in the helix conformation is statistically higher than in the coil conformation (*p*-value < 0.05) in Fig. 4(g). Comparison with Fig. 4(c) reveals that the RNA binding residues are overpredicted among the helical residues and underpredicted among coils. The main point, which is reflected in Fig. 4(f) and (h) that quantify the relations between predicted DNA-/RNA-binding and predicted secondary structure, is that the relations that we identify using the predictions replicate the relations observed based on experimental data. This includes the highest relative fraction of DNA-/RNA-binding residues in the coil conformation, followed by helix and strand, and the fact that the three pairwise differences are statistically significant (*p*-value < 0.05).

**Fig. 5:**
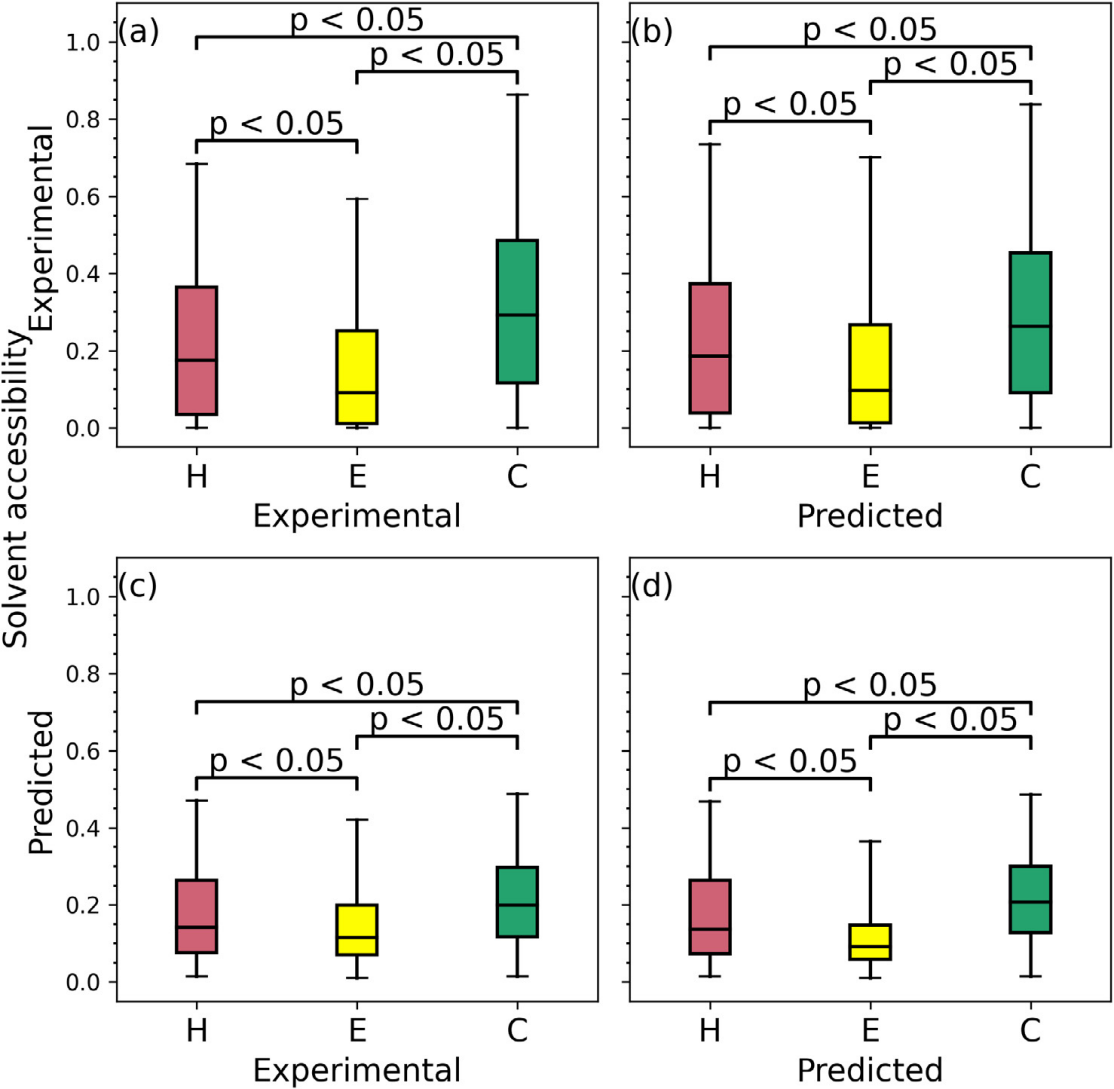
Relation between solvent accessibility and secondary structure on the part of the low-similarity test dataset that has the corresponding experimental data. Panel (a) shows experimental values of solvent accessibility and secondary structure. Panel (b) summarizes experimental solvent accessibility and predicted secondary structure. Panel (c) compares predicted solvent accessibility against the experimental secondary structure. Panel (d) shows relation between predicted solvent accessibility and predicted secondary structure. The color-coded box plots (red for helix, yellow for strand, and green for coil) represent distributions of the solvent accessibility values using the 5th (bottom whisker), 25th, 50th, 75th and 95th (top whisker) percentiles. We assess significance of differences in the solvent accessibility values between residues that have different secondary structures (helix vs. coil, helix vs. strand and strand vs. coil) using the *t*-test if the underlying data are normal; otherwise, we use the Wilcoxon signed-rank test; we test normality with the Anderson-Darling test at the 0.05 significance. The corresponding *p*-values are included at the top of the box plots. (For interpretation of the references to color in this figure legend, the reader is referred to the web version of this article.)

The remaining experiment focuses on the relation between the secondary structures and solvent accessibility (Fig. 5). Fig. 5(a) reveals that residues in the coil conformation have statistically significantly higher solvent accessibility compared to the helical residues (*p*-value < 0.05), which in turn have significantly higher solvent accessibility compared to the strand residues (*p*-value < 0.05). This agrees with several works that show that coils/loops are usually more solvent exposed than helices, while strands are more frequently buried [117], [118]. Fig. 5(b) compares the experimental solvent accessibility values between residues grouped based on their putative secondary structures. We observe that it closely resembles the relations from the experimental data from Fig. 1(a), including the statistical significance of the differences. Similarly, Fig. 5(c) summarizes the relation between putative solvent accessibility and experimental secondary structure. We note that while the putative solvent accessibility is characterized by a narrower range of values compared to the native/experimental values these predictions still accurately reflect relations with the secondary structure. Finally, Fig. 5(d) shows that the relation between predicted solvent accessibility and predicted secondary structure replicates the relation between the experimental values, including the highest solvent accessibility values for coils followed by helices and strands and the fact that the three pairwise differences (helix vs. coil, helix vs. strand and strand vs. coil) are statistically significant (*p*-value < 0.05). This suggests that the solvent accessibility and secondary structure predictions can be used both individually and together to accurately reflect the native data. Furthermore, this provides the final piece of support for our overarching claim that the relations between different types of native structural/functional characteristics of amino acids are accurately replicated by the corresponding predictions.

## 4 Summary

The last few decades have seen an influx of sequence-based predictors of the residue-level annotations of protein function and structure. Popular examples include methods that predict intrinsic disorder, secondary structure, solvent accessibility, and protein and nucleic acid interaction sites. Numerous assessments and comparative surveys were done to catalogue and compare these methods [1], [7], [13], [15], [29], [32], [38], [39], [40], [41], [42], [43], [44], [45], [46], [47], [48], [49], [50], [51], [52], [53]. These studies assist users in selection of the most accurate or the most suitable tools, measure progress over time and help in formulating future research directions. However, methods that focus on a given prediction target are typically analyzed and evaluated in isolation from the other types of methods, while these diverse predictors are used to analyze the same proteins. To the best of our knowledge, relationships between different predicted structural and functional features have never been tested against the corresponding experimental data. This motivated our systematic study that explored relations between all pairs of the key residue-level characteristics including secondary structure, solvent accessibility, intrinsic disorder and nucleic acids binding. We study complementarity in two scenarios. First, when combining predictions of the nucleic acid binding residues generated by the structure- and disorder-trained predictors. Second by investigating whether relations among a comprehensive collection of six pairs of the characteristics that are present in the experimental data are accurately reflected by the corresponding predictions. These analyses rely on a large and consistent dataset of human proteins that share low similarity (<25%) to the training data used to develop the underlying predictors.

Our major finding is that the predictions accurately replicate relations between solvent accessibility, secondary structure, interaction sites and intrinsic disorder that are measured using experimental data. This suggests that the various predictions can be used together to accurately reflect the native data, extending results of the past studies that show that they produce accurate results individually. This paves the way to utilize multiple different residue-level predictors together to gain insights concerning protein structure and function and to develop new bioinformatics systems. A few recent examples include analysis of the SARS-CoV2 proteome that applies predictions of protein and nucleic acids interacting residues and intrinsic disorder [21]; development of a database of membraneless organelles that describes associated proteins using predictions of disorder, pi-pi contacts and nucleic acid binding residues [119]; system that predicts protein structure quality using the putative solvent accessibility and intrinsic disorder [120]; and a methodology that predicts deleterious single amino acid variations by relying on the putative secondary structure, intrinsic disorder, and coiled-coil regions [121].

Moreover, motivated a recent finding concerning prediction of the protein-binding residues [58], we show that the structure-trained and the disorder-trained predictors of DNA-binding and RNA-binding residues produce complementary results. We find that combining their outputs using a neural network produces predictions that significantly outperform the results that they generate individually. This suggests that they should be used together to maximize the accuracy of the prediction of nucleic acid binding residues. We provide the resulting neural network model as a convenient webserver at https://biomine.cs.vcu.edu/servers/MetaNucBind/.

## CRediT authorship contribution statement

**Balint Biro:** Data curation, Investigation, Methodology, Validation, Writing – original draft. **Bi Zhao:** Data curation, Investigation, Methodology, Validation, Writing – original draft, Writing – review & editing, Software, Supervision. **Lukasz Kurgan:** Conceptualization, Methodology, Validation, Writing – original draft, Writing – review & editing, Project administration, Resources, Supervision, Funding acquisition.

## Declaration of Competing Interest

The authors declare that they have no known competing financial interests or personal relationships that could have appeared to influence the work reported in this paper.
