# Supplementary material for: Complementarity of the residue-level protein function and structure predictions in human proteins
Source: Comput Struct Biotechnol J. 2022 May 6;20:2223–34. doi: 10.1016/j.csbj.2022.05.003 (PMC9118482; doi:10.1016/j.csbj.2022.05.003)
Supplement: Supplementary data 1 [file mmc1.pdf]

Supplement for "Complementarity of the residue-level protein function and structure predictions in human proteins"

Balint Biro[1,2#], Bi Zhao[2#*], Lukasz Kurgan[2*]

[1]Institute of Genetics and Biotechnology, Hungarian University of Agriculture and Life Sciences, Gödöllő, Hungary

[2]Department of Computer Science, Virginia Commonwealth University, Richmond, Virginia, United States

[#]co-first authors (these authors have contributed equally)

*Corresponding authors: Bi Zhao (zhaob4@vcu.edu) and Lukasz Kurgan (lkurgan@vcu.edu)

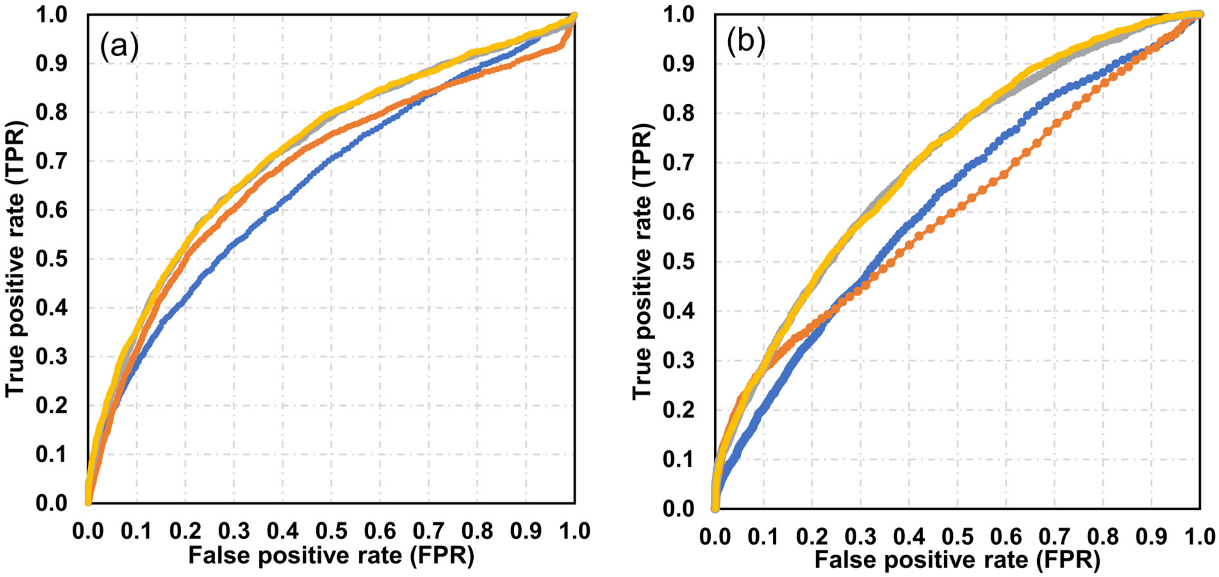

Supplementary Figure S1. Receiver operating characteristic (ROC) curves for predictions of (a) DNA-binding and (b) RNA-binding on the test dataset. The blue line is the performance of the disorder-trained DisoRDPbind. The orange is for the structure-trained DRNApred. The grey is based on the average normalized score of DisoRDPbind and DRNApred. The yellow is for the MetaNucBind predictor.

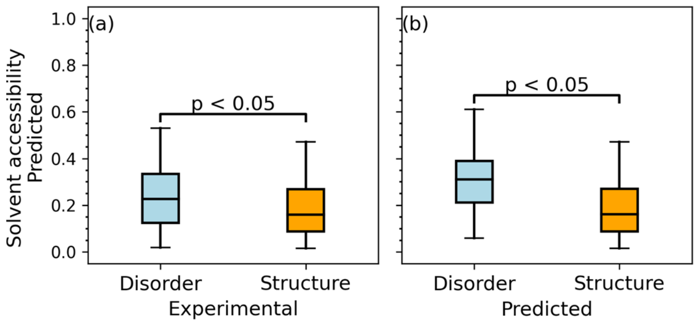

Supplementary Figure S2. Relation between solvent accessibility and intrinsic disorder on the part of the low-similarity test dataset that has the corresponding experimental data. Panel (a) summarizes predicted solvent accessibility against the experimental intrinsic disorder. Panel (b) shows relation between predicted solvent accessibility and predicted intrinsic disorder. The color-coded box plots (blue for disorder; yellow for structure) represent distributions of the solvent accessibility values using the 5th (bottom whisker), 25th, 50th, 75th and 95th (top whisker) percentiles. We assess significance of differences in the solvent accessibility values between residues that are disordered or structured using the *t*-test if the underlying data are normal; otherwise, we use the Wilcoxon signed-rank test; we test normality with the Anderson-Darling test at the 0.05 significance. The corresponding *p*-values are at the top of the box plots.
